# G-CSF treatment in decompensated liver disease: a double-edged sword?

**DOI:** 10.1007/s12072-022-10379-8

**Published:** 2022-09-02

**Authors:** Cornelius Engelmann, Thomas Berg

**Affiliations:** 1 Department of Hepatology and Gastroenterology, Campus Virchow-Klinikum, Charité-Universitaetsmedizin Berlin, Berlin, Germany; 2 Berlin Institute of Health (BIH), Berlin, Germany; 3 Liver Failure Group, Institute for Liver and Digestive Health, University College London, Royal Free Campus, London, UK; 4 Division of Hepatology, Department of Medicine II, Leipzig University Medical Center, Liebigstr. 20, 04103 Leipzig, Germany

Granulocyte colony-stimulating factor (G-CSF) exhibits regenerative and immunomodulatory properties, therefore, representing an attractive therapeutic approach for patients with advanced liver disease. There was almost no substance that has provided similarly remarkable results in end-stage liver disease as G-CSF, and consequently it was already considered a possible standard therapy for these patients. Published in 2012, Garg et al. showed in a small single-center trial with in total of 47 patients that the administration of G-CSF with a dose of 5 µg/kg s.c. and 12 injections over a period of 26 days improved the 60-day survival of patients with acute-on-chronic liver failure (ACLF) from about 30% to almost 70% [1]. The treatment success was attributed to an improved immune cell function, fewer infectious complications as well as higher numbers of CD34+ stem cells in the liver potentially facilitating its recovery from injury. A comparable improvement in survival was shown when the effect of G-CSF was investigated in further studies either in patients with acute alcoholic hepatitis [2, 3] or with decompensated cirrhosis [4, 5]. In severe acute alcoholic hepatitis, the 90-day mortality rate declined from more than 70% after standard of care to about 20% when patients were treated with G-CSF (with a dose of 10 µg/kg/day) in two randomised single-center trials and in both studies G-CSF therapy resulted in fewer severe infections [2, 3]. Even in decompensated cirrhosis that does not show features of ACLF, short-term therapy with G-CSF has demonstrated its potential on improving patients’ survival in several randomised trials [4, 5]. Among them, two studies are especially worth mentioning as they enrolled a high number of patients. Prajapati et al. randomized in total of 253 patients to receive either a short term, high-dose G-CSF therapy (300 µg twice daily over a period of 5 days) or standard medical therapy. The cumulative 6-month survival of 79% when G-CSF was given was significantly higher than 68% (*p* = 0.025) after standard medical therapy with more deaths caused by a severe infection in the latter group [5]. De et al. tried to maximise the therapeutic effect by applying long-term G-CSF therapy given every 3 months in 5 days cycles for a total of 12 months to patients with decompensated liver cirrhosis. G-CSF improved substantially the disease course with an increased 12-month survival of 74% compared to 42% in the control group (*p* < 0.001) and fewer episodes of infections [4].

However, the view that G-CSF may be a potential new standard therapy in patients with decompensated cirrhosis with or without ACLF has been questioned by the results of two European multicentre studies that failed to confirm the positive effects previously observed. The REALISITC trial evaluated whether G-CSF as a monotherapy or in combination with hematopoietic stem cell reinfusion acted as a disease-modifying therapy for patients with compensated liver cirrhosis and a MELD score of 11 to 15.5. In total 81 patients were randomly assigned to one of the three treatment arms and G-CSF was given high-dose of 15 µg/kg for 5 days. None of the experimental therapies was able to exhibit a beneficial effect on survival or liver function [6]. The GRAFT study, a large multicentre trial, was originally initiated with the aim to confirm the efficacy of G-CSF in ACLF. By using the identical study protocol as Garg et al. [1] this study failed to show improvement in the 90-day transplant-free survival, which was 34.1% and 37.5% in the G-CSF and standard of care arm [7].

The study by PMID: 35322373 presented in the current issue of Hepatology International tried to add more evidence on the safety and efficacy of G-CSF long-term treatment in patients with decompensated cirrhosis. The same group which published the first study with multiple cycles of G-CSF in decompensated cirrhosis [4] presented a single-center follow-on study where they applied the identical study design as well as the same in- and exclusion criteria to a subsequent cohort recruited between May 2019 and June 2020. They randomized 70 patients with decompensated cirrhosis defined by hospital admission due to ascites, hepatic encephalopathy or variceal bleeding but no evidence of ACLF according to the APASL and/or EASL EF-CLIF criteria. G-CSF was given at a dosage of 5 µg/kg every 12 h subcutaneously for 5 days and these treatment cycles were repeated every three months for up to 12 months and in total four cycles. The primary endpoint was the overall survival after 1 year. After 12 months of G-CSF therapy, the survival in the G-CSF arm was not significantly better with 87.9% compared to 66.7% (*p* = 0.053) for those receiving standard medical therapy. None of the patients was transplanted. Patient treated with G-CSF improved their Child–Pugh score, most likely due to an improvement of ascites, whilst the MELD score was unaffected, generally adding to the persistent conflicting results between the single-center trials and recent multicentre trials. In addition, G-CSF treatment reduced the number of SBP infections from 39.4% to 12.1% whilst non-SBP infections were not different. The rational to prolong the treatment with G-CSF in a cohort with lower short-term mortality than patients with ACLF to maximise its pro-regenerative and immune-modulatory capacities seems plausible and indeed, the resolution of ascites and the low number of SBP infections was likely to be the consequence of these effects.

However, potential shortcomings of the current study design need to be discussed in the context of the mixed treatment response with an improvement of Child–Pugh score and reduced SBP rate on the one hand and no impact on patients’ outcome in terms of transplant-free mortality on the other hand. It can be argued that this is mainly a consequence of insufficient sample size, which means that consistent results could have been achieved with a larger number of patients. This point highlights a potential general concern not only with the current study but with most of the overall existing body of studies on the use of G-CSF in patients with advanced liver disease, where the study design, particularly the small sample size and lack of external validation due to the single-centre study design, does not allow us to answer questions about subgroups who may or may not benefit from G-CSF therapy. It rather increases the risk of a selection bias and overestimation of the actual effect size. Assuming that G-CSF may have its indication in certain subgroups of patients with end-stage liver disease a collaborative work including individual patient data from all studies performed so far would help identifying markers to predict treatment response for G-CSF and to decipher the heterogeneity of patients among the trials.

Among the pleotropic effects of G-CSF, the modification of immune cell function is one of those of particular importance when considering potential beneficial effects in patients with decompensated liver disease and ACLF [8]. These patients are at high risk of infections due to their impaired capacity for adequate pathogen response. The G-CSF effect on immune cell function may help reducing the risk of infection, which is the most important trigger for ACLF development and is associated with high mortality. Indeed, based on the observation that G-CSF reduces the risk of infections in some trials [1, 4] immune modulation and strengthening of pathogen response may be one central beneficial pathomechanistic element during treatment with G-CSF, although data is not consistent throughout the trials [7].

When critically reviewing the current literature it becomes apparent that G-CSF may have several and in part opposing effects on immune cell phenotypes and functionality depending on the cellular microenvironment and disease state. Fostering the recruitment and generation of immunomodulatory cells by G-CSF to reduce inflammation and prevent infections may be desirable in end-stage liver disease and in fact, mobilisation of CD4+ CD25+ CD39+ regulatory T cells was associated with better outcome [9]. In addition, high numbers of circulating myeloid and plasmacytoid dendritic cells were linked to improved survival in ACLF [10]. It would be worth exploring to what extent the cellular composition of the bone marrow defines the capacity to mobilise distinct subsets of regulatory immune cells thereby defining treatment response to G-CSF in liver disease. Low numbers of hematopoietic cells in the bone marrow identified a subgroup of patients with poor regenerative capacity and prognosis [11, 12] although it remains unclear to what extent these progenitors bolster regenerative activities directly or rather by interaction with other cell types, e.g. mesenchymal stem cells.

G-CSF, however, not simply induces unidirectional, beneficial effects in individual cell subsets but may also lead to immune cell changes, which can be detrimental. Cell subsets mobilised by G-CSF can exhibit a more pro-tumorigenic phenotype as shown for Ly6G^+^Ly6C^+^ neutrophils, which enhance tumours growth in models of metastatic tumours [13]. This sort of more aggressive immune cell phenotype was also observed after treatment with G-CSF in models of endotoxin-driven liver disease. When injecting LPS into rats after partial hepatectomy, G-CSF pre-treatment increased the inflammatory response, death rates and microcirculatory disturbances [14]. G-CSF not only upregulated LPS binding protein in the bone marrow and liver tissue but also caused excess mortality along with TLR4 overexpression in the liver in models of LPS-related liver failure [15]. Hence, G-CSF can also be considered to act as a sensitizer to endotoxin-inducing tissue injury. This may be deleterious in certain forms of advanced stages of liver cirrhosis and especially ACLF, in which the endotoxin TLR4 signalling axis and high levels of TLR4 ligands were shown to drive disease progression [16, 17]. And indeed, the GRAFT trial reported in total seven severe adverse reactions related to the G-CSF therapy, most of them showing features of aggravated inflammatory response, and 3 out of 4 patients died due to these events [7]. In other trials where G-CSF was shown to improve survival in ACLF described drug-related adverse events to be mild and non-life threatening [1, 18]. Therefore, the potential deleterious effect of G-CSF is less prominent in the clinical setting.

Is G-CSF a double-edged sword and does the question of whether patients should be treated with G-CSF or not depend mainly on when in the course of the disease G-CSF is administered? It seems plausible that patients with recent ACLF onset and high-grade systemic inflammation are less likely to benefit from an additional recruitment of neutrophils and monocytes by G-CSF therapy. However, a considerable number of patients remain in a status of persistent organ dysfunction and lack of recovery after the resolution of the first ACLF event [19, 20]. These patients are at high risk of secondary infections and death due to immune dysfunction and additional incapacity to regenerate from organ failure [21]. G-CSF comprises a pro-regenerative capacity, which boosts proliferation also in progenitor and parenchymal cells, and immune-stimulatory capacity to maintain an adequate pathogen response [22]. This combinatory effect is warranted, in particular for the above-mentioned cohort. The fact that inflammation per se may prevent adequate regeneration put high demands on the adequate selection and characterisation of the ideal treatment population [23]. It seems logical that patients with active infections or patients shortly after disease onset where systemic inflammation is the predominant pathomechanistic feature may be at risk of suffering G-CSF-related side effects but late disease stages with immune paralysis and non-recovery can represent a population with a desirable risk profile.

Further studies addressing both, disease-related and treatment-related aspects, will be needed to understand in which clinical scenario G-CSF may act beneficially and to refine the target population accordingly. The art will be to decipher the exact mechanisms by which G-CSF acts in the context of the different stages of liver disease and to identify those who may potentially benefit from its immune-modulatory or pro-regenerative capacities or both. To this end, harmonisation of the current definitions of decompensated liver cirrhosis and ACLF, which are still characterised by relevant clinical heterogeneity, would be a fundamental prerequisite. We also need to better understand whether long-term administration with multiple cycles of G-CSF, as shown in the current study, is preferable to short-term treatment to stabilise neutrophil and monocyte function and reduce the long-term risk of infection in patients with end-stage liver disease. Further clinical trials need to investigate whether, particularly in clinical conditions where there is a risk of endotoxin-induced organ dysfunction, such as ACLF and acute alcoholic hepatitis, both, regeneration and inflammation can be positively influenced synergistically by a combination of G-CSF with an anti-inflammatory drug, which could expand the safety and efficacy as well as the therapeutic window for the use of G-CSF. Other combinatory or sequential treatment approaches where G-CSF was complemented by other stimulatory compounds such as erythropoietin [24] or growth hormone [25] reported similar favourable outcomes. However, these approaches may not broaden the potential target population by targeting additional pathomechanisms but rather add to the pro-regenerative effect of G-CSF itself.
